# Using airway resistance measurement to determine when to switch ventilator modes in congenital diaphragmatic hernia: a case report

**DOI:** 10.1186/s12887-020-02258-8

**Published:** 2020-08-03

**Authors:** Sasagu Kimura, Katsuaki Toyoshima, Tomoaki Shimokaze, Rikuo Hoshino

**Affiliations:** 1 Department of Neonatology, Kanagawa Children’s Medical Center, 4-138-2 Mutsukawa Minamiku Yokohama, Yokohama, Kanagawa 232-2351 Japan; 2 Department of Pediatrics, Yamaguchi University Graduate School of Medicine, Ube, Japan

**Keywords:** Conventional mechanical ventilation, Extra-corporeal membrane oxygenation, High frequency oscillatory ventilation, Oxygenation index, Persistent pulmonary hypertension of the newborn

## Abstract

**Background:**

Congenital diaphragmatic hernia is a deficiency of the fetal diaphragm resulting in herniation of the abdominal viscera into the thoracic cavity. The best method of respiratory management of congenital diaphragmatic hernia is unclear, but high frequency oscillatory ventilation is often used as the initial ventilator mode for severe congenital diaphragmatic hernia. When it becomes impossible to maintain the pre-ductal saturations, the timing of successful switching of the ventilation mode from high frequency oscillatory ventilation to conventional mechanical ventilation remains unclear. Herein, we reported two cases in which airway resistance measurements based on pulmonary function tests were used for making the decision to switch the ventilator mode from high frequency oscillatory ventilation to conventional mechanical ventilation in patients with left isolated congenital diaphragmatic hernia.

**Case presentation:**

Two 0-day-old infants with congenital diaphragmatic hernia were admitted to our hospital. In both patients, high frequency oscillatory ventilation was started initially, and the levels of saturation gradually rose within a few hours after birth. After 24 h of high frequency oscillatory ventilation, the level of saturation decreased, and the dissociation of pre-ductal and post-ductal saturation re-occurred. The respiratory-system resistance was 515 and 403 cmH_2_O·kg/L/s, respectively. Because the respiratory-system resistance was elevated, we decided to change the ventilator mode from high frequency oscillatory ventilation to conventional mechanical ventilation. After switching to conventional mechanical ventilation, the patients’ heart rate and saturation increased immediately.

**Conclusions:**

In patients with congenital diaphragmatic hernia, resistance levels of > 400 cmH_2_O·kg/L/s may indicate high airway resistance and suggest greater alveolar vibration attenuation. When respiratory-system resistance reaches over 400 cmH_2_O·kg/L/s, it may be an optimal time for switching from high frequency oscillatory ventilation to conventional mechanical ventilation.

## Background

Congenital diaphragmatic hernia (CDH) is characterized by a deficiency of the fetal diaphragm resulting in herniation of the abdominal viscera into the thoracic cavity [1]. The overall population incidence ranges from 1.7 to 5.7 per 10,000 births [1]. Pulmonary hypoplasia of the ipsilateral and/or contralateral sides, depending on the size of the defect and pulmonary hypertension, is the major problem leading to mortality and morbidity, and is associated with one of the poorest outcomes in neonates [2].

The selection of initial ventilator settings for CDH patients is critical to ensure adequate ventilation while minimizing barotrauma. Regarding the best ventilator strategy for newborns with CDH, there is no obvious superiority of one ventilator mode compared to others. The American Pediatric Surgical Association (APSA) recommendation suggests that there is more clinical experience with conventional ventilation strategies that minimize barotrauma and allow permissive hypercapnia in the CDH population. Thus, conventional strategies are suggested as the preferred method for ventilation. Initial ventilator modes, such as intermittent mandatory ventilation, are recommended for positive inspiratory pressure (PIP) < 25 cmH_2_O, positive end-expiratory pressure (PEEP) =3–5 cmH_2_O, pre-ductal SaO_2_ > 85%, and PCO_2_ < 60 mmHg [3]. High frequency oscillatory ventilation (HFOV) may be considered when PIP is > 25 cm H_2_O or SaO_2_ is < 85% after optimization of other clinical parameters [3]. The use of HFOV versus conventional ventilation in infants with congenital diaphragmatic hernia was investigated in an international randomized clinical trial (VICI trial) of the CDH Euro consortium comparing conventional mechanical ventilation (CMV) and HFOV as the initial ventilation method. The initial settings for CMV were a PIP of 20–25 cmH_2_O, and a PEEP of 3–5 cmH_2_O, with a ventilator rate of 40–60/min [4]. The initial settings for HFOV were a mean airway pressure (MAP) of 13–17 cmH_2_O, a frequency of 10–12 Hz, and a delta P of 30–50 cmH_2_O, depending on chest wall vibration [4]. There are studies comparing HFOV and CMV as initial ventilator methods, but few studies have examined the effectiveness of HFOV as an initial ventilator in CDH cases. Therefore, there is a little data on HFOV for the management of CDH [5]. Additionally, the VICI trial is the only multicenter randomized controlled trial indicating the optimal time to switch the ventilator mode from HFOV to CMV. Here, we report two cases in which the pulmonary function test was successfully used for deciding the timing of switching the ventilator mode from HFOV to CMV.

## Case presentation

### Case 1

**Fig. 1:**
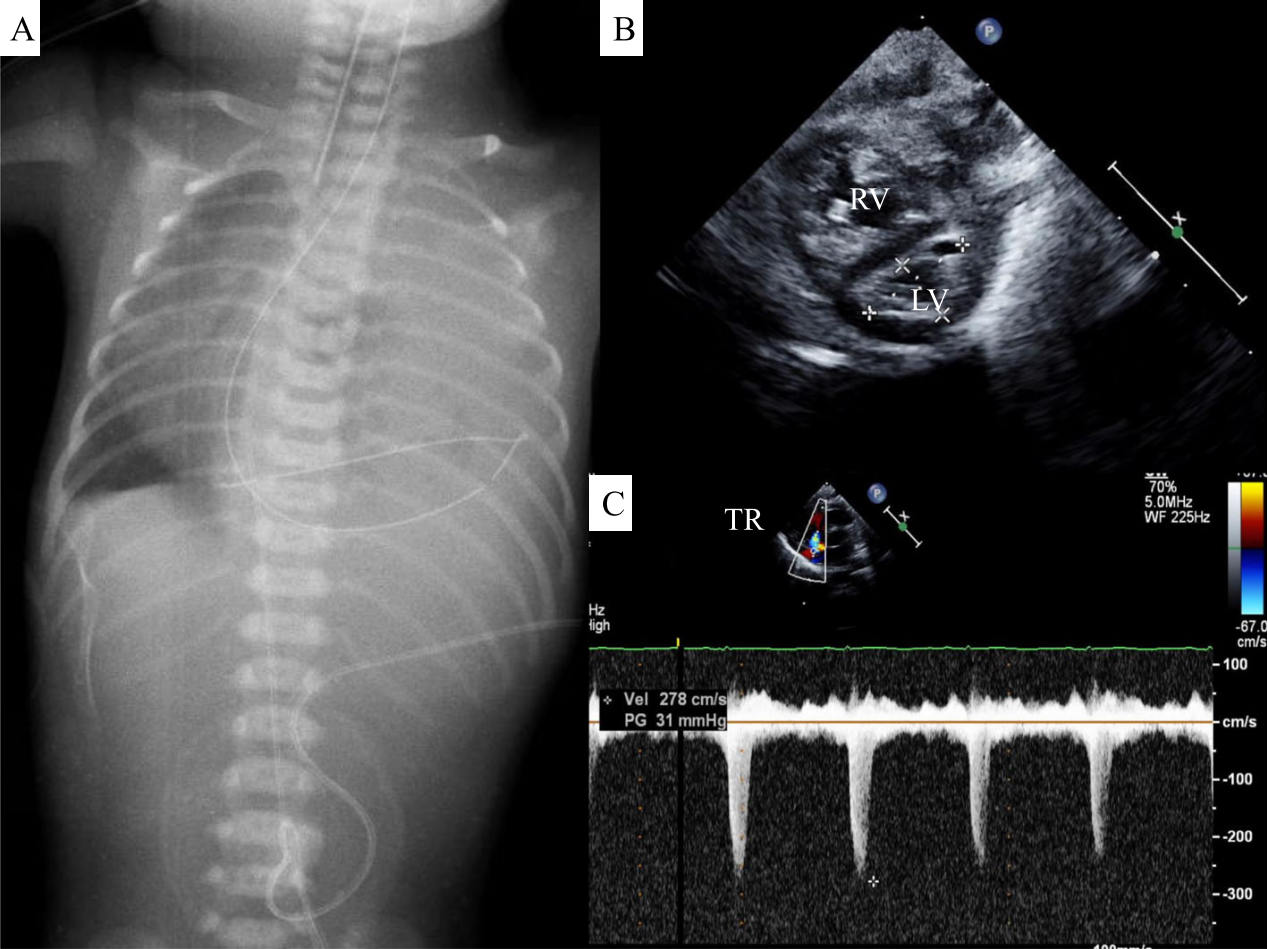
Clinical findings of case 1. **a**. The intestinal tract herniated into the left thoracic cavity, and left lung hypoplasia was observed. The mediastinum and heart are offset to the right. **b**. The right ventricle expanded causing the left ventricle to be overwhelmed. **c**. Tricuspid regurgitation was moderate, and the estimated right ventricular pressure was 36 mmHg. The body’s blood pressure at that time was 54/38 (45) mmHg. LV: left ventricle. RV: right ventricle. TR: Tricuspid regurgitation

The patient was a 0-day-old male neonate with left isolated CDH. The diagnosis of CDH was made prenatally, and 46, XY, der(21)t(1;21)(q42.3;q22.2) was diagnosed via examination using the amniotic fluid. Fetal ultrasonic testing showed a lung thorax transverse area (LT) ratio of 0.10. He was born at full term, 38 weeks and 2 days, through vaginal delivery in the delivery room. The birth weight was 2105 g. After birth, intubation (uncuffed endotracheal tube, 3.5 mm, Smiths Medical, USA) was performed immediately. After admission to the neonatal intensive care unit (NICU), the piston HFOV (calliope-alpha, Metran, Japan) was started as the initial ventilation. At the initial settings, the MAP was 14 cmH_2_O, the stroke volume (SV) 20 ml, and the frequency 12 Hz. Inhaled nitric monoxide (iNO) was used at 20 ppm because of the persistent pulmonary hypertension of the newborn (PPHN) (Fig. 1). Fourteen hours after birth, PPHN increased and Lipo-prostaglandin E1 (Lipo-PGE1) was administered to keep the arterial duct open and maintain blood flow to the pulmonary artery. After administration of Lipo-PGE1, the level of saturation was increased both pre-ductally and post-ductally, and the saturation was kept around 95% for the next 30 h. The level of saturation decreased, and the dissociation of pre-ductal and post-ductal saturation re-occurred 44 h after birth. Increasing the iNO concentration and MAP was ineffective. Blood gas analysis revealed an alveolar-arterial oxygen difference (AaDO_2_) of 500 Torr, and oxygenation index (OI) of 20. The PaO_2_/FiO_2_ (P/F) ratio was 58.8 (Fig. 3a–c), which required considering the introduction of extra-corporeal membrane oxygenation (ECMO) to save the patient’s life. Before introducing the ECMO, we inspected the pulmonary function tests results. The static pulmonary function was measured by occlusion method using ARFEL III (Aivision, Japan), which adapted a Fleischer type of pneumotachograph. Because under HFOV, pulmonary function tests cannot be performed accurately, we changed ventilator mode from HFOV to CMV, and used muscle relaxant to rule out the effects of spontaneous breathing just before running the test. The occlusion valve operation was measured with 4–5 breaths to confirm reproducibility. The respiratory-system compliance (Crs) level was 0.29 ml/cmH_2_O/kg and the respiratory-system resistance (Rrs) level was 515 cmH_2_O·kg/L/s. Because the Rrs level was elevated, we made the decision to shift the ventilator mode from HFOV to CMV at the ventilator settings of PIP 20 cmH_2_O, PEEP 5 cmH_2_O, and ventilator rate of 40/min. After switching to CMV, the patient’s heart rate rose from 159 bpm to 169 bpm, and saturation rose from 89 to 96% immediately. Thirty minutes after switching to CMV, blood gas tests revealed that the AaDO_2_ dropped to 275 and the OI to 7.3, while the P/F ratio increased (Fig. 3a–c). At 55 h after birth, the operation for repairing the diaphragm was performed. The patient was then discharged from our hospital at 1 month of age with an uneventful course of treatment.

### Case 2

**Fig. 2:**
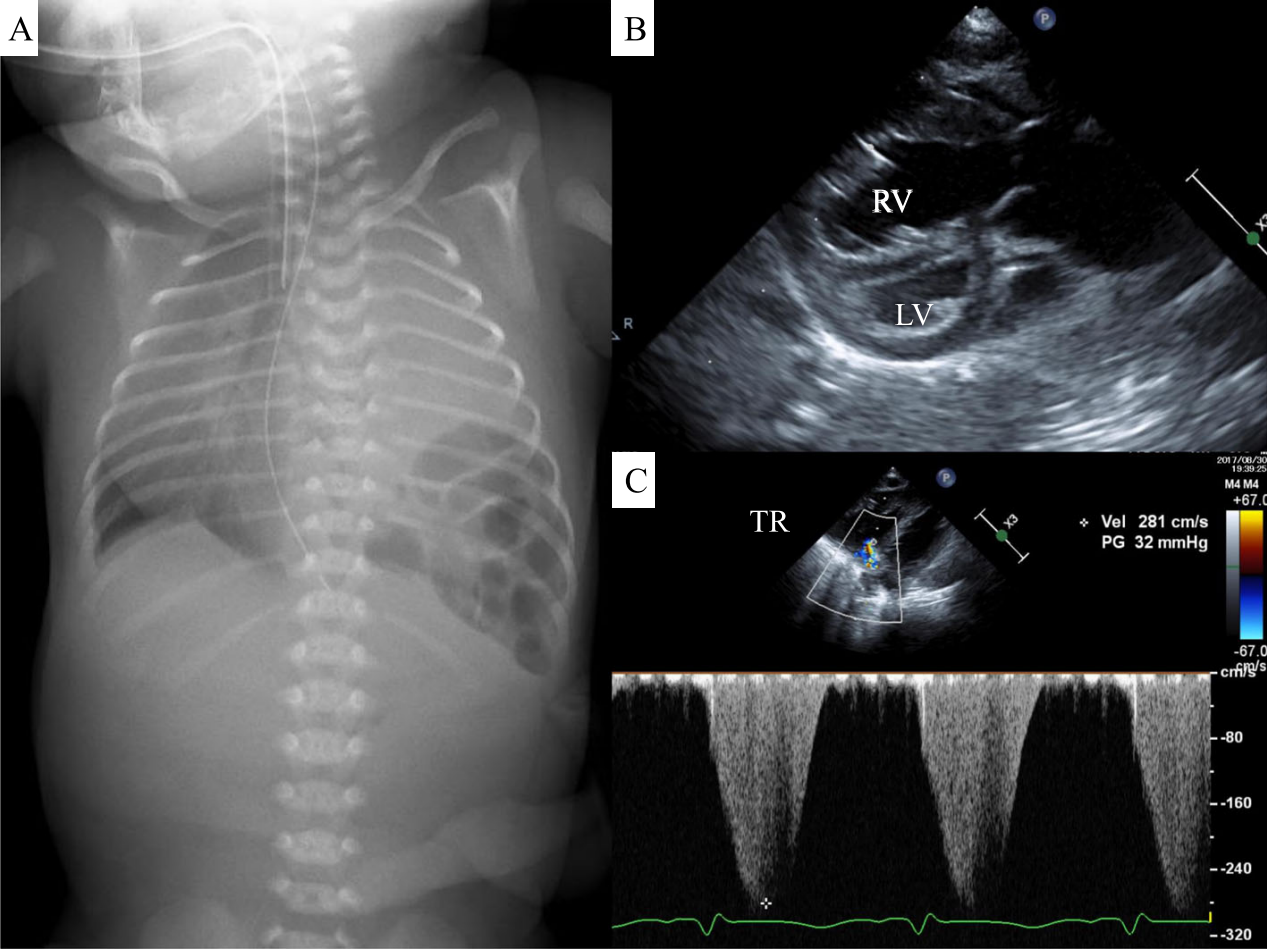
Clinical findings of case 2. **a**. The intestinal tract herniated into the left thoracic cavity, and left lung hypoplasia was observed. The mediastinum and heart are offset to the right. **b**. The right ventricle expanded overwhelming the left ventricle. **c**. Tricuspid regurgitation was moderate and the estimated right ventricular pressure was 37 mmHg. The body’s blood pressure at that time was 49/38 (43) mmHg. LV: left ventricle. RV: right ventricle. TR: Tricuspid regurgitation

**Fig. 3:**
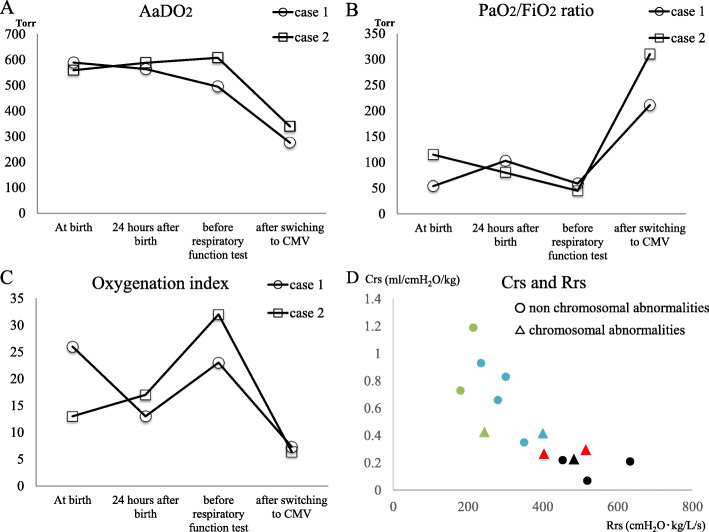
Clinical course of blood gas analysis evaluation and cases of pulmonary function tests measured at our institute. **a**. In both case 1 and case 2, the AaDO_2_ remained high from birth until the change to CMV. After changing to CMV, AaDO_2_ decreased significantly in both cases. **b**. The PaO_2_/FiO_2_ ratio remained below 100 before changing to CMV but improved after the change to CMV. **c**. In case 1, the OI decreased 24 h after birth, but increased before switching to CMV. The OI declined after the change to CMV. In case 2, the OI gradually increased after birth, and rose to a level that required consideration of ECMO before the pulmonary function test. After switching to CMV, the OI dropped prominently. **d**. The values of Crs and Rrs measured at our institute. Black dots are death cases and red dots are the cases described in this article using HFOV as the initial ventilation. Blue dots are the cases whose lives were saved using HFOV as the initial ventilation, which was not changed to CMV. Green dots are the cases in whom initial ventilation was CMV, which was not changed to HFOV. The triangle points are cases with chromosomal abnormalities, and dots are the cases without chromosomal abnormalities. CMV: conventional mechanical ventilation. ECMO: extra-corporeal membrane oxygenation. Crs: respiratory-system compliance. Rrs: respiratory-system resistance. HFOV: high frequency oscillatory ventilation

The patient was a 0-year-old male neonate with left isolated CDH. The diagnosis of CDH was made prenatally. Fetal ultrasonic testing showed a LT ratio of 0.16. The liver was not in the thoracic cavity, and the stomach was in the abdomen. He was born at full term, 39 weeks and 5 days, through caesarean section. The birth weight was 3145 g. After birth, intubation (uncuffed endotracheal tube, 3.5 mm, Smiths Medical, USA) was performed immediately, and piston HFOV (calliope-alpha, Metran, Japan) was started as the initial ventilation method. The patient’s MAP was 15 cmH_2_O, SV 35 ml, and frequency 12 Hz for the initial settings. iNO was used at 20 ppm due to PPHN (Fig. 2). The level of saturation gradually rose to 98%. Facial features suggested Pallister-Killian syndrome, and a chromosome test revealed mos 47, der(12)(pter→q10)[13]/46, XY[17]). At 30 h after birth, the level of post-ductal saturation decreased to 85% and dissociation of pre-ductal and post-ductal saturation occurred. Increasing iNO and alkalizing was ineffective. Blood gas analysis showed an AaDO_2_ of 608 Torr, and an OI of 32. The F/P ratio was 45, which indicated the introduction of ECMO (Fig. 3a–c). Pulmonary function tests at this time revealed that the level of Crs was 0.26 ml/cmH_2_O/kg, and Rrs was 403 cmH_2_O·kg/L/s. The level of Rrs was clearly rising, thus, we decided to switch the ventilator mode from HFOV to CMV at the ventilator settings of PIP 25 cmH_2_O, PEEP 5 cmH_2_O, and ventilator rate of 40/min. After switching the mode, the heart rate rose from 97 bpm to 160 bpm and post-ductal saturation rose from 92 to 97% immediately. Thirty minutes after switching to CMV, blood gas showed that AaDO_2_ dropped to 339 Torr, and OI dropped to 6.4, while the P/F ratio rose (Fig. 3a–c). Five days after birth, the diaphragm was repaired via a surgical procedure. The patient was then discharged from our hospital at the age of 2 months with an uneventful course of treatment.

## Discussion and conclusion

We report two cases of CDH in whom we used pulmonary function tests to determine when to switch mechanical ventilation from HFOV to CMV. There are a few indications on when to switch ventilator modes; however, it is unclear in which cases switching the ventilator mode from HFOV to CMV will be successful. Former studies, such as the VICI trial, have provided possible indications for switching the ventilator mode from HFOV to CMV. The criteria for switching the ventilator mode in the VICI trial are one or more of the following: inability to maintain pre-ductal saturations above 85% or post-ductal saturations above 70%; an increase in CO_2_ to greater than 65 mmHg, despite optimization of ventilator management; PIP > 28 cmH_2_O; MAP > 17 cmH_2_O; or an OI of longitudinal evaluation ≧ 40 [4]. In our cases, the condition worsened as the levels of PaO_2_/FiO_2_ and AaDO_2_ decreased, and OI increased. These findings did not meet the switching criteria of the VICI trial. In similar cases, evaluation of airway resistance may be able to suggest when to switch the ventilator mode with successful outcomes.

The vibrational diffusion of HFOV is attenuated by the flow-dependent resistance and inertial resistance of the endotracheal tube and airway. The vibrations in the alveoli are attenuated compared to mouth vibrations, reducing the risk of lung damage [6, 7]. Therefore, HFOV has been used as a gentle ventilation to minimize barotrauma for meconium aspiration syndrome and CDH [8, 9]. High airway resistance causes more attenuation of the vibrational diffusion in the alveoli, which is an important problem that needs to be addressed for HFOV. In both of our cases, the Rrs was over 400 cmH_2_O·kg/L/s. At our institute, we perform the pulmonary function tests for diaphragmatic hernia, but Rrs is not always in an elevated range. The following data is from our institute from cases in whom the ventilator mode was not changed (Fig. 3d). The number of cases using HFOV as the initial ventilator was five and using CMV as the initial ventilator was three, respectively. Statistical analyses were not performed due to the small number of cases. When the severity was mild and HFOV was used, the Rrs was in a range of 235–400 cmH_2_O·kg/L/s, and the median of Rrs was 301 cmH_2_O·kg/L/s. In the cases that used CMV as the initial ventilator, the Rrs range was 179–243 cmH_2_O·kg/L/s, and the median was 214 cmH_2_O·kg/L/s. In diaphragmatic hernia cases, Rrs of more than 400 cmH_2_O·kg/L/s may indicate high airway resistance and suggests greater attenuation of alveolar vibration. Although, the cause of increased airway resistance in diaphragmatic hernia is not well understood, it is thought that compression and twisting of the airways due to the escape of organs into the thoracic cavity may be the cause. According to our cases, Rrs over 400 cmH_2_O·kg/L/s may be an indicator for switching the ventilator mode from HFOV to CMV.

A limitation of our study is that both cases had a chromosome abnormalities (46, XY, der (21)t(1;21)(q42.3;q22.2) and mos 47, der(12)(pter→q10)[13]/46, XY[17]). Chromosomal abnormalities, including complete or mosaic chromosome aneuploidies, large chromosome deletion/duplications, and complex chromosome rearrangements identifiable by karyotype are present in 10–35% of CDH cases [10]. The possibility that the structural abnormality in the lung tissue was associated with the respective chromosomal abnormalities cannot be denied. The values of Crs and Rrs measured in our institute are shown in Fig. 3d. Patients with chromosomal abnormalities tend to have low compliance and high resistance. However, there is a similar tendency in severe cases such as death cases. It may be applicable to severe cases regardless of chromosomal abnormalities.

Moreover, we used uncuffed endotracheal tube because we predicted that CDH would require long-term ventilation management. In order to reduce the air leak, we have selected the larger outer diameter of endotracheal tube. Additionally, the exact leak rate was unknown because the air leak was not displayed on the ventilator (calliope-alpha, Metran, Japan) that we used in these patients. The presence or absence of leak was judged by clinical symptoms such as chest movement. Also, since the respiratory function test with ARFEL III evaluates the pressure and the flow rate, it is possible to predict the qualitative evaluation, such as the decrease in the baseline value, if there is a leak. If there was a leak, neck was slightly pressed to correspond to the leak. Therefore, our results might be applicable when using an uncuffed endotracheal tube only.

In conclusion, the optimal timing of successfully switching the ventilator mode from HFOV to CMV remains unclear. However, pulmonary function tests of airway resistance may be a useful tool for this decision-making. A further study is therefore necessary to determine the utility of airway resistance, based on pulmonary function tests, for switching the ventilator mode from HFOV to CMV in CDH patients.

## Data Availability

Not applicable.

## Abbreviations

AaDO_2_: Alveolar-arterial oxygen difference
CDH: Congenital diaphragmatic hernia
CMV: Conventional mechanical ventilation
Crs: Respiratory-system compliance
ECMO: Extra-corporeal membrane oxygenation
HFOV: High frequency oscillatory ventilation
iNO: Inhaled nitric monoxide
Lipo-PGE1: Lipo-prostaglandin E1
LT: Lung thorax transverse area
MAP: Mean airway pressure
NICU: Neonatal intensive care unit
OI: Oxygenation index
PPHN: Persistent pulmonary hypertension of the newborn
Rrs: Respiratory-system resistance
SV: Stroke volume
